# Editorial and Miscellaneous

**Published:** 1866-10

**Authors:** 


We learn that Dr. Samuel Logan, formerly Demonstrator of Anatomy and Adjunct Professor of Surgery in the Medical College of South Carolina, has been elected to the chair of Anatomy in the Medical College of Virginia, in place of A. E. Peticolas, resigned.

